# Evaluation of a multiprofessional, nonsurgical obesity treatment program: which parameters indicated life style changes and weight loss?

**DOI:** 10.1186/s40337-017-0144-4

**Published:** 2017-05-15

**Authors:** Pjanic, Roland Müller, Markus Laimer, Niels Hagenbuch, Kurt Laederach, Zeno Stanga

**Affiliations:** 1 Department of Diabetes, Endocrinology, Clinical Nutrition and Metabolism, University of Bern and University Hospital of Bern, 3010 Bern, Switzerland; 2 Private statistician, Spiez, Switzerland; 3 Department of Department of Visceral Surgery and Medicine, University of Bern and University Hospital of Bern, 3010 Bern, Switzerland

**Keywords:** Obesity, Behavioral therapy, Multiprofessional intervention, Life style behavioral change, Weight loss

## Abstract

**Background:**

According to the current evidence, behavior modifications are an effective part of a non-surgical multiprofessional obesity treatment program (MOTP). The purpose of the present study was to report changes in weight as well in psychological variables during a one year MOTP. We aimed to identify the associations of emotional state and patients’ emotion regulation skills with weight change.

**Methods:**

Prospective interventional study. Data of participants attending the one year obesity treatment in either a group or individual structured MOTP were analyzed. Weight, BMI (Body Mass Index) and measures on psychosomatic variables, emotion regulation skills, affective state, shame and guilt were collected at baseline, after three months and after one year. Mixed-effects models were used for the statistical analysis of BMI.

**Results:**

We included 238 patients at baseline (t1), 234 after three months (t2) and 179 after one year (t3). A drop in BMI measurements of at least 5% was observed in 20.6% of participants at t2 and 41.4% of participants at t3. After three months, participants showed significant improvements in the following psychosomatic variables: somatisation (*p* < 0.001), interpersonal sensitivity (*p* < 0.001), emotion regulation skills (*p* < 0.01), and attention to emotions (*p* < 0.05). Most of the improvements could be maintained after one year. BMI reduction was associated with a positive change in emotions, improvements in emotion regulation skills, and a reduction of depressive symptoms, disgust and shame.

**Conclusions:**

The results indicate that the assessment and treatment of psychological aspects like depression, emotion regulation skills, body awareness, and acceptance should be a vital part of an interdisciplinary MOPT.

**Trial registration:**

Ethical approval for the present study was obtained from the Bern Kantonal Ethics Committee (KEK-Bern-Study Nr 258/14), Bern, Switzerland.

## Plain English Summary

### Context

Obesity is an increasing health problem worldwide and we need to know the best methods to treat it. It is standard practice that professionals with different therapeutic backgrounds guide people to change their lifestyle habits. Our research examines which psychological components of a multidisciplinary obesity treatment are associated with a reduction in BMI.

### What does the trial do?

Our patients were enrolled in a year-long program with a team of dieticians, psychologists, body awareness therapists and medical doctors. We were interested in results on weight, wellbeing and handling of feelings.

### More information

We gathered data of 238 patients who took part in the obesity treatment program at the university hospital of Berne between 2010 and 2014. We measured:

- weight
- wellbeing
- handling of feelings

### Impact and outcomes

Our results show a decrease in weight and improvements in wellbeing, especially mood and shame. Patients handling of their feelings was associated with weight loss, especially if they improved in:

- handling of strongly negative feelings
- attention to feelings
- attention to their body

We show that psychological coaching and body awareness therapy may be relevant to obesity treatment. Future studies should examine the different influences further.

## Background

According to the WHO, two billion people worldwide are overweight, of whom a third are obese. This represents a major risk for non-communicable diseases and for global mortality [1]. In 2012, 31% of the adult Swiss population was overweight, and an additional 10% was obese. Over the last 20 years, Switzerland has experienced a considerable increase in the prevalence of overweight and obesity in the adult population growing from 30.3 to 41.3% [2]. From a societal viewpoint, the estimate of the total Swiss economic burden of overweight and obesity and associated diseases (comorbidities) has almost tripled over the past ten years, from CHF 2.6 billion in 2002 to CHF 8 billion in 2012. This represents 12.4% of the total Swiss healthcare expenses [3].

The current obesity epidemic appears to be the result of an interaction between environment, behavioural aspects, and genetic susceptibility [4]. An imbalance between energy consumption and expenditure is the central reason for this condition, which is a major risk for health [1, 4]. Besides these medical-biological and financial problems there are also psychological burdens. As shown in several studies, the obese population suffers from a higher ratio of psychopathologic disorders, in particular anxiety, dysfunctional emotion regulation and major depression [5–8]. It is well known that emotional dysregulation may lead to emotional eating, which plays a central role in reducing negative mood in people with obesity [9–11].

Therefore, it is very important that a comprehensive multiprofessional obesity treatment program (MOTP) implies not only lifestyle modification, such as limited energy intake and regular physical activity, but also cognitive psychotherapy focussing on emotion regulation and encouraging behavioral changes [12]. An individualized, multi-component, multidisciplinary treatment is important for successful weight loss, since a single intervention generally does not meet the needs for all individuals with obesity, and has been shown to be less effective [4, 13]. In a Cochrane review, Shaw et al. reported that psychotherapy, in combination with hypocaloric diet and physical exercise, is effective and leads to greater weight reduction compared with diet and/or exercise alone [13]. Although we know that MOTPs with a psychological component are effective, there is still a major lack in understanding the factors influencing efficacy of psychological interventions on weight loss because long-term outcome studies are still missing [13]. However, research provides evidence that addressing emotion regulation skills is more important than dealing with the patients’ general wellbeing in reducing emotional eating as a help for weight reduction [8].

The primary aim of the current study was to report longer term outcome effects of our interdisciplinary MOTP regarding change in weight, emotional state and emotion regulation skills. Further objectives of the study were to evaluate the impact of psychological factors (i.e., emotions and emotion regulation skills) on weight change, and to define important psychological treatment factors within the MOTP.

## Methods

### Design

Single centre, prospective interventional study at the Obesity Outpatient Clinic (OOC) of the Division of Diabetes, Endocrinology, Clinical Nutrition and Metabolism, University Hospital of Bern, Switzerland. The study was conducted between April 2010 and July 2014.

### Patients

We included obese out-clinic patients with a body mass index (BMI) ≥30 kg/m^2^, women and men older than 18 years, able to speak and understand German, who attended the individual or group nonsurgical MOTP at the OOC.

### Procedure

Participants with obesity were recruited from the OOC within the outpatient consultations. The participants were mostly referred to the MOTP by their primary care physicians (general practitioner). The costs of the treatment were paid by the patients’ insurance. At the beginning the participants signed a therapy-contract in which the agreements and responsibilities were defined. In order to assess the patient’s suitability for the individual or group therapy program, a personal interview with both a psychologist and a dietician took place at the beginning of the MOTP. The indication for the individual treatment program was given if there were psychosocial and motivational reasons or personal circumstances (e.g., job, family), which hindered the participation in the group program.

Anthropometric data and psychometric questionnaires were assessed before therapeutic intervention, after three months, and after one year of the MOTP.

### Description of the group and individual MOTP

#### Group program

The group program consisted of two parts. The first part lasted 12 weeks, with the first 6 weeks being the most intensive treatment phase. During weeks 1 through 6, patients had a weekly 90 minutes session in cognitive behavioral therapy (CBT), three 90 min sessions in nutrition counseling, a weekly two hour session in body-awareness therapy, and two 60 min exercise sessions to improve physical activity levels. During weeks 7 to 12, patients received weekly alternating CBT session or nutrition counselling. Also, a weekly 60 minutes sports session was added. The CBT and the nutrition counselling followed a manualized structure for the three month. The CBT addressed defining realistic weight goals, self-monitoring of dysfunctional eating habits, analyzing one’s own behavior, setting up flexible behavioral goals, education on emotion regulation, and training of emotion regulation skills. The nutrition counseling included information on a balanced diet, addressing the combined importance of fat, proteins and carbohydrates, and focusing on a reduction of self imposed prohibition of certain foods. The physical activity modalities included water gymnastics, Nordic walking, and weight lifting in the hospital’s fitness center. After three months, an individual evaluation session took place with the psychotherapist and the medical assistant under supervision of a trained senior doctor. In this session, each patient received an evaluation of test results and their progress was evaluated. Patients then switched to the second part of the group treatment. In this phase the transfer into everyday life was a central focus. The patients had a monthly session alternatingly between CBT and nutrition counselling. In these sessions, progress and obstacles to their goals were discussed. Additionally, psychological topics and nutritional advice from the first 12 weeks intensive treatment were repeated. The physical activities had to be organized by the patients themselves as part of the transfer process into everyday life. As a part of the monthly CBT sessions, activity levels were monitored, and patients were motivated to keep up with their new training routines and higher activity levels. After one year, an individual evaluation session took place again, and patients had the possibility to receive further psychological and medical assistance if needed.

#### Individual program

Over the course of the year, the individual program consisted of monthly scheduled sessions with a dietician and the presence of a trained senior doctor every three months. On a voluntary basis, patients in the individual program could also attend a course in body-awareness therapy over 8 weekly sessions and/or a psychological coaching over 7 weekly sessions where CBT elements from the group program were provided. Both optional treatments were held in groups. Similar to the group program, patients had the possibility to receive further psychological and medical assistance if required.

### Measurements

Data were collected at three measurement points (baseline, t1; after three-months, t2; and after one year, t3), and included anthropometric measurements (weight, height, BMI: defined as the body weight divided by the square of the body height, and is universally expressed in units of kg/m^2^), psychological questionnaires, and the socio-demographic records. Socio-demographic data were not for all patients collected.

The following questionnaires were applied:

#### EMO-Check

In order to evaluate the emotional state, the EMO-Check (emotional inventory) was used [14]. Originally developed in Switzerland, the self-reporting questionnaire includes 50 items assessing emotions like stress, anxiety, anger, sadness, depression, shame, coping, and positive and negative affect.

#### SEK-27

The EMO-Check also includes the SEK-27 questionnaire (self-evaluation of emotional skills) including 27 items concerning handling of emotions with the following scales: attention to own emotions (e.g., “During the last week I paid attention to my feelings”), emotional clarity (e.g., “During the last week I could clearly tell what I was feeling”), awareness of bodily sensations (e.g., “During the last week I had good body awareness regarding my feelings”), understanding of emotions (e.g., “During the last week I knew why I felt the way I was feeling”), acceptance of the emotions (e.g., “During the last week I was sure to be able to tolerate negative emotions”), resilience (e.g., “During the last week I could do what I had intended to do even while experiencing negative feelings”), self-support (e.g., “During the last week I remained myself in tough situations”), willingness of confrontation (e.g., “During the last week I knew I could influence my feelings”), emotion regulation (e.g., “During the last week I could accept negative feelings”), and a total score. The questionnaire further consists of two items to evaluate the presence of disgust and shame. The internal consistency and concept validation among clinical and normative population for both questionnaires have been documented as satisfactory to excellent [14]. In an intervention study, Berking et al. demonstrated that incorporating interventions, which directly target general emotion-regulation skills, may improve the effectiveness of psychotherapeutic interventions [15].

#### SCL 90-R

The symptom checklist (SCL 90-R) [16] is a well-established questionnaire with 90 items measuring subjectively perceived impairment. It is multidimensional and consists of the scales somatization (e.g., “In the past 4 weeks, how much were you distressed by headaches”), obsessive-compulsive symptoms (e.g., “In the past 4 weeks, how much were you distressed by having to do things very slowly to ensure correctness”), interpersonal sensitivity (e.g., “In the past 4 weeks, how much were you distressed by the feeling that people are unfriendly or dislike you”), aggressiveness/hostility (e.g., “In the past 4 weeks, how strongly were you distressed by having urges to break or smash things”), anxiety (e.g., “In the past 4 weeks, how much were you distressed by spells of terror or panic”), depression (e.g., “In the past 4 weeks, how much were you distressed by feeling no interest in things”), paranoid ideation (e.g., “In the past 4 weeks, how much were you distressed by feeling that most people cannot be trusted”), phobic anxiety (e.g., “In the past 4 weeks, how much were you distressed by feeling afraid to go out of the house alone”), and psychoticism (e.g., “In the past 4 weeks, how much were you distressed by having thoughts that were not your own”).

### Statistical analysis

Analyses were conducted using IBM SPSS Statistics for Windows, version 21.0 [17] (released 2012, Armonk, NY: IBM Corp.) and R version 3.3.2 [18]. To compare the patients included in the analyses with those who were excluded, we performed independent *t*-tests to examine initial age, initial BMI and initial questionnaire scores, as well as a Chi-squared test to compare gender.

Due to missing values at different time points, variables measured repeatedly over time (i.e., BMI, SCL 90-R, SEK-27, and EMO-Check) were examined by a linear mixed-effects model using the lme4 package in R [19]. BMI was considered the response variable, while the respective questionnaires, program, age at baseline, and gender entered the model as covariates. A scalar single random effects term, i.e., the patients, was included (random-intercept model). The parameters were estimated using the maximum-likelihood method. Confidence intervals are based on the profiled deviance. In all analyses, diagnostic plots of the residuals revealed no violations of the assumptions of normality and homoscedasticity. The set-up of the separate models was pre-specified based on psychological expert knowledge on emotion regulation, and all sub-items of a respective questionnaire were analyzed together. No variable-selection procedures were applied. In the first model, we included psychological stress, measured by the 10 items of the SCL 90-R questionnaire. In the second model, we included emotion regulation skills (the 9 items of the SEK-27 questionnaire). In the third model, we included the 7 items of the EMO-Check measuring specific emotions, and in the fourth model, we included the 4 items of the EMO-Check measuring state and trait affect. The fifth model included the 2 screening scales for disgust and shame of the EMO-Check.

## Results

### Participants and sample characteristics

Between April 2010 and July 2014 a total of 307 obese individuals were recruited. Out of these, three individuals were excluded because of a switch from the group to the individual treatment program. An additional three individuals were excluded because of change to a surgical procedure. No individuals switched from the individual to the group treatment program. Forty-nine of the remaining individuals were excluded because the psychological questionnaires were incomplete (the most frequent reason was lack of motivation), and 14 individuals were excluded due to missing weight data at one or more time points. Thus, the present analysis focused on 238 individuals, 150 (63.0%) participants of the group treatment program and 88 (37.0%) participants of the individual program. One hundred-ninety one (80.2%) were female and 47 (19.8%) were male. The average BMI at the beginning of treatment was 39.6 kg/m^2^ (standard deviation 6.2 kg/m^2^), and the mean age was 41.4 years (SD 12.3 years). The BMI was comparable for both programs over the three measurement points (Table 1). The majority of the patients finished elementary school and were married.

**Table 1:** Demographics and BMI

Demographics	Data
Gender, *n* (%)	
Male	47 (19.8)
Female	191 (80,2)
Age in years, *mean* (SD)	
Group treatment program	42.5 (12)
Individual treatment program	39.4 (12.7)
Treatment program, *n* (%)	
Group	150 (63)
Individual	88 (37)
Education, *n* (%)	
Special needs school	2 (0.8)
School	79 (33.2)
Apprenticeship	23 (9.7)
University/higher education	20 (8.4)
Others	2 (0.8)
Missing	112 (47.1)
Marital status, *n* (%)	
Single	50 (21)
Married	90 (37.8)
Divorced	13 (5.5)
Partnership	9 (3.8)
Missing	76 (31.9)
BMI in kg/m^2^ at t1, *mean* (SD)	
Group treatment program	40.1 (6.3)
Individual treatment program	38.9 (6.1)
BMI in kg/m^2^ at t2, *mean* (SD)	
Group treatment program	38.7 (6.3)
Individual treatment program	38.3 (6.2)
BMI in kg/m^2^ at t3, *mean* (SD)	
Group treatment program	37.8 (6.5)
Individual treatment program	37.3 (6.7)

No significant differences in gender or initial BMI were observed between patients included or excluded from the analysis, but they differ significantly in age (*p* <0.01), in initial SCL 90-R score for obsessive compulsiveness (*p* <0.05), and in the total of positive symptoms (*p* ≤0.00). Those who were excluded were younger, had a lower score in obsessive-compulsive scale, and a lower score in total positive symptoms than those, who were included. Because of the small sample sizes, a differential analysis between the excluded subgroups was not feasible.

### Internal consistency of the questionnaires EMO-Check and SCL 90-R

In our study, internal consistency indicated by cronbach’s alpha, (for the first part of the EMO-Check) varies over the measurement points as follows: at t1, *α* = 0.66–0.93; at t2, *α* = 0.66–0.95; at t3, *α* = 0.63–0.95. For the second part of the questionnaire (SEK-27), internal consistency varies as follows: at t1, *α* = 0.71–0.81; at t2, *α* = 0.69–0.85; at t3, *α* = 0.63–0.87. The internal consistency for the symptom checklist (SCL 90-R) varies over the measurement points as follows: at t1, *α* = 0.75–0.89; at t2, *α* = 0.84–0.92; at t3, *α* = 0.85–0.93.

### Weight loss assessed by the BMI and changes in psychological variables

**Fig. 1:**
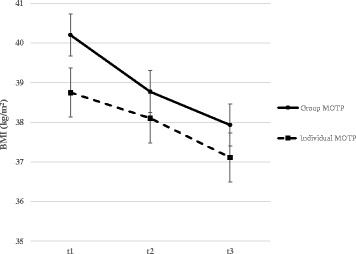
BMI over time by group. Note: The plot shows the average BMI in kg/m2 over time split by group. The vertical bars indicate the standard errors of the mean. In practice, 3 kg weight loss corresponds approximately to 1 unit decrease in BMI

A statistically significant drop in BMI and weight was observed throughout the one-year treatment. After three months, patients showed a median weight loss of 2.5 kg (IQR 0.7–5.1 kg), and after one year a median weight loss of 4.2 kg (IQR 1.1–8.6 kg). The mean BMI over the three measurement points was at t1, 39.6 kg/m^2^; at t2, 38.5 kg/m^2^; and at t3, 37.7 kg/m^2^. The BMI decreased significantly between baseline t1 and three months t2 (*p* < 0.05) and between baseline t1 and one year t3 (*p* <0.05) (see Table 2). These results are presented separately for the individual and group program in Fig. 1.

**Table 2:** BMI and Scales of Symptom checklist 90 at each timepoint

Characteristics	Baseline		3 months			*p*	Baseline		12 months		
	Mean	SD	Mean	SD	*n*		Mean	SD	Mean	SD	*n*
BMI	39.6	6.2	38.5	6.2	234	<0.05	39.6	6.3	37.7	6.6	179
SCL 90-R											
Somatization	59.1	10.9	54.6	11.2	130	<0.001	57.0	12.3	54.8	11.1	30
Obsessive-compulsive symptoms	57.6	10.6	54.7	11.5	131	<0.01	56.0	11.5	53.1	11.7	32
Interpersonal sensitivity	59.2	11.6	55.4	11.3	131	<0.05	56.7	10.3	51.4	10.9	32
Depression	60.4	10.4	56.1	11.6	131	<0.05	56.9	11.7	52.1	10.7	32
Anxiety	55.7	10.8	53.5	10.4	131	0.008	55.6	11.4	51.5	12.0	32
Aggressiveness	55.3	10.4	53.1	10.2	131	0.018	52.6	12.1	51.0	11.8	32
Phobic anxiety	53.4	10.3	52	10.4	131	0.076	53.3	10.1	50.8	10.4	32
Paranoid ideation	54.9	10.9	53	10.9	131	0.018	54.3	9.7	49.7	10.8	31
Psychoticism	56.4	10.7	54.3	10.2	131	0.011	56	12.1	52.7	11.4	31
Global severity index	59.1	10.8	55.3	11.6	131	<0.05	56.6	12.7	53.3	11.7	32

Footnote: The *p*-values are from paired t-tests

The change in BMI did not differ significantly between the group and individual program (*p =* 0.08) nor between gender (*p* = 0.72). After three months, the weight data of 233 patients revealed that 20.6% of individuals had lost at least 5% of their initial body weight, and after a year, the weight data of 179 patients showed that 41.4% lost at least 5% of their initial weight.

Altogether, the psychological questionnaires revealed several significant changes over time. In the SCL 90-R, all variables except “phobic anxiety” and “aggressiveness” showed a significant decrease after three months (*p* < 0.05) (Table 2).

After one year, similar results persisted, except for the scale “aggressiveness” (*p* <0.05). Table 3 shows details of the paired t-Tests for the psychological variables of EMO-Check-Emotions and SEK-27. After three months, patients showed significant higher attention to emotions, significantly higher scores in the regulation of emotions, acceptance and resilience Furthermore, all SEK-27 measurements of emotion showed significant changes after three months except screening of guilt and disgust. At one year, only acceptance showed a significant increase compared to the beginning of the program. Comparing Emo-Check scales from beginning to one year, depression and shame decrease significantly whereas overall coping emotions show a significant increase. Further, the positive emotions scale and positive emotions scale total improved significantly from beginning to one year.

**Table 3:** Scales of EmoCheck-Emotions and SEK at each timepoint

Characteristics	Baseline		3 months			*p*	Baseline		12 months			*p*
	Mean	SD	Mean	SD	*n*		Mean	SD	Mean	SD	*n*	
Attention to the emotions	6.7	2.8	7.8	2.5	130	**<0.05**	6.7	2.4	7.3	2.7	30	0.168
Bodily awareness of feelings	7.7	2.7	8	2.5	129	0.188	7.9	2.5	8.3	2.6	31	0.328
Clarity about emotions	8.3	2.5	8.2	2.5	130	0.440	8.8	2.2	9.1	2.4	31	0.426
Understanding of emotions	8.3	2.5	8.2	2.6	127	0.657	8.7	2.3	9	2.6	31	0.430
Emotion regulation	6.5	2.5	7.2	2.4	127	**0.005**	7	2.1	7.3	1.8	31	0.305
Acceptance of the emotions	7.4	2.6	8	2.5	125	**0.019**	7.7	2.8	8.7	2.3	31	**0.013**
Resilience	6.7	2.8	7.4	2.8	130	**0.006**	7.6	2.7	8.3	2.4	31	0.090
Self-support	7.4	2.6	7.8	2.6	129	0.209	7.4	2.1	7.6	2.4	31	0.540
Willingness of confrontation	7.2	2.7	7.5	2.3	130	0.266	7.8	2.1	8.1	2.3	30	0.491
Stress	5.3	3.3	4.5	3.2	133	**0.006**	4.7	2.6	4.1	3	31	0.213
Anxiety	4.1	3.2	3.5	3.2	133	**0.024**	3.2	2.6	3	2.8	31	0.641
Anger	3.7	2.9	3.2	2.8	133	**0.029**	2.7	1.8	2.8	2.5	31	0.873
Sadness	4.7	3.3	3.8	3	133	**<0.05**	3.3	2	3.5	2.7	31	0.743
Depression	3.6	3.5	2.7	3.2	133	**0.001**	2.2	2.6	1.3	2.1	31	**0.041**
Shame	2.5	2.7	1.9	2.2	133	**0.002**	1.9	2.3	1	2.2	30	**0.031**
Coping emotions	24.6	8.2	26.2	9.2	133	**0.025**	26	6.6	29.3	4.8	31	**0.001**
Positive affect	22.4	7.3	24.1	8.8	133	**0.009**	23.8	6.6	27.2	4.4	31	**0.006**
Negative affect	12.1	8.6	10.3	8.7	133	**0.012**	8.5	5.6	8.5	6.5	31	1.000
Positive affect total	54	17.8	58.4	19.7	133	**0.002**	56.2	15.6	63.6	11.2	31	**0.002**
Negative affect total	30.4	20.6	24.9	20	133	**<0.05**	23	13.8	19.1	16.5	31	0.115
Disgust	0.7	1.1	0.5	1	131	0.139	0.6	1.2	0.3	0.9	30	0.133
Guilt	1	1.3	0.8	1.2	132	0.078	0.6	0.9	0.6	0.8	30	0.873

Footnote: The *p*-values are from paired t-tests. Numbers in bold indicate significance

### Influence of emotions and emotion regulation on BMI

The mixed-effects models for BMI were calculated separately for each questionnaire. Program, age at baseline, and gender were included as covariates. For the symptom checklist (SCL 90-R), somatization and interpersonal sensitivity increased BMI significantly by 0.05 kg/m^2^ and 0.06 kg/m^2^, respectively, with each unit. None of the other scales had any significant influence on BMI in this model (Table 4).

**Table 4:** SCL 90-R estimates of BMI

	BMI	
Estimates	Coefficient	95% CI
SCL 90-R (382 observations in 228 patients)		
Intercept	35.03	31.34; 38.72
Program (individual vs. group)	−1.13	−2.82; 0.55
Age	−0.06	−0.12; 0.01
Gender (men vs. women)	0.82	−1.21; 2.85
**Somatization**	**0.05**	**0.01; 0.09**
Obsessive-compulsive symptoms	−0.02	−0.07; 0.02
**Interpersonal sensitivity**	**0.06**	**0.01; 0.11**
Depression	0.02	−0.03; 0.08
Anxiety	−0.04	−0.09; 0.00
Aggressiveness	0.01	−0.03; 0.04
Phobic anxiety	0.01	−0.03; 0.05
Paranoid ideation	0.01	−0.04; 0.05
Psychoticism	0.02	−0.02; 0.07
Global severity index	0.01	−0.10; 0.12

Footnote: Results of a mixed-effects model. At each of the three time points, the BMI was modelled by the actual values of SCL 90-R scales whereas age entered the model as baseline value. The 95% confidence intervals are stated, lines in bold indicate significant influence on BMI

The SEK-27 scales “attention to emotions” and “awareness of bodily sensations” reduce BMI significantly by −0.15 kg/m^2^ and −0.16 kg/m^2^, respectively, with each unit. The scale “emotional clarity” increases BMI by 0.36 kg/m^2^ with each unit. None of the others scales had any significant influence on BMI in this model (Table 5).

**Table 5:** Results of 4 mixed-effects models (SEK-27 and EMO-Check estimates of BMI)

	BMI	
Estimates	Coefficient	95% CI
SEK-27 (353 observations in 216 patients)		
Intercept	42.36	38.95; 45.77
Program (individual vs. group)	−1.2	−2.98; 0.58
Age	−0.06	−0.13; 0.01
Gender (men vs. women)	1.07	−1.10; 3.25
**Attention to the emotions**	**−0.15**	**−0.28; −0.02**
**Bodily awareness of feelings**	**−0.16**	**−0.31; −0.00**
**Clarity about emotions**	**0.36**	**0.16; 0.56**
Understanding of emotions	0.04	−0.15; 0.24
Emotion regulation	−0.12	−0.31; 0.08
Acceptance of the emotions	−0.13	−0.32; 0.06
Resilience	−0.004	−0.17; 0.16
Self-support	0.06	−0.10; 0.22
Willingness of confrontation	−0.03	−0.16; 0.10
EMO-Check emotions (385 observations in 228 patients)		
Intercept	42.36	38.85; 45.87
Program (individual vs. group)	−0.99	−2.73; 0.75
Age	−0.07	−0.13; 0.00
Gender (men vs. women)	1.11	−1.00; 3.22
Stress	−0.03	−0.16; 0.09
Anxiety	−0.08	−0.22; 0.07
Anger	−0.01	−0.16; 0.14
Sadness	−0.11	−0.25; 0.04
**Depression**	**0.24**	**0.08; 0.41**
Shame	0.13	−0.02; 0.29
Coping emotions	−0.02	−0.08; 0.03
Affect items of EMO-Check (386 observations in 229 patients)		
Intercept	42.25	38.78; 45.75
Program (individual vs. group)	−1.02	−2.72; 0.68
**Age**	**−0.07**	**−0.14; −0.00**
Gender (men vs. women)	1.08	−0.99; 3.14
Positive affect	−0.07	−0.16; 0.01
**Negative affect**	**−0.1**	**−0.19: −0.02**
Positive affect total	0.02	−0.03; 0.06
**Negative affect total**	**0.06**	**0.02; 0.10**
Screening items of EMO-Check (369 observations in 222 patients)		
Intercept	41.79	38.72; 44.86
Program (individual vs. group)	−1.12	−2.86; 0.63
**Age**	**−0.07**	**−0.14; −0.00**
Gender (men vs. women)	1.12	−0.98; 3.22
**Disgust**	**0.37**	**0.07; 0.66**
Shame	0.06	−0.20; 0.31

Footnote: Results of four separate mixed-effects models. At each of the three time points, the BMI was modelled by the actual values of SEK-27 and EMO-Check scales, respectively, whereas age entered the model as baseline value. The 95% confidence intervals are stated, lines in bold indicate significant influence on BMI

For the EMO-Check scales, “depression” increases BMI by 0.24 kg/m^2^ with each unit, “negative affect” reduces BMI by −0.10 kg/m^2^ with each unit, whereas “negative affect total” increases BMI by 0.06 kg/m^2^ with each unit. In this model, age reduces BMI significantly by −0.07 kg/m^2^ with each year. Of the two screening items, disgust reduces BMI significantly by 0.37 kg/m^2^ with each unit. Also, in this model, age reduces BMI significantly by −0.07 kg/m^2^ with each year. In a model where BMI and age were log-transformed and items of SCL 90-R, EMO-Check and SEK-27 were arcsine square root transformed, also anxiety in the SCL 90-R, stress and shame in the EMO-Check were significant.

## Discussion

In this cohort the participants were found to have reduced BMI and made a positive change in emotions and emotion regulation, reduced depression and shame. After three months a fifth of the individuals (20.6%) and after one year nearby half of the remaining individuals (41.4%) achieved a weight loss of at least 5% compared to the baseline bodyweight, a weight reduction which, according to Racette et al. [4] is considered as effective. Furthermore, individuals improved in experienced emotional distress and affective state. Regarding the reduction in depression levels, the patients had similar effects to those demonstrated in patients after bariatric surgery [20]. In fact, we observed a continuous decrease from the baseline to one year.

Feelings of shame have a negative impact on eating behavior, especially on binge eating [21, 22]. One important origination for shame is related to obesity stigmatization and the persistent discrimination, which leave individuals feeling ashamed as well more uncertain in the interpersonal contact. Westermann et al. [23] showed that social exclusion of obese individuals compared to normal-weight controls causes a stronger increase in shame. Therefore, the authors concluded that psychological interventions should focus on shame-related emotional distress. Our data reveal a significant reduction in shame over the whole treatment course of one year. Our data indicate that psychological factors addressed through intensive treatment consisting of body awareness treatment and regular psychological therapy, may have had a contribution in achieving this goal.

In our statistical model shame had a relevant influence not only on weight loss, but also on the current emotional state, especially the negative affect. Reducing shame might reduce dysfunctional eating patterns in patients with obesity, which leads to a weight reduction. Our results show that alongside with a reduction in negative affect a reduction in body weight may be achieved. This fact can be explained by the concept of emotional eating [24, 25].

In the current study patients also improved their skills in emotion regulation. They showed higher levels of attention to emotions, acceptance and resilience after three months, with acceptance remaining an important factor in the regulation of emotions at one-year measures. According to the literature, psychological treatment on resilience and acceptance in particular, deliver promising positive effects in obese individuals [26, 27]. It has been demonstrated by Lillies et al. [26] that even a short educational intervention with focus on acceptance and mindfulness in patients with obesity showed an improvement of patients’ dealing with stigma, stress, overall and weight related acceptance [26]. In our MOTP, the included body awareness treatment addresses dealing with stress, overall and weight related acceptance, as well as training in general body awareness and attention to emotions. It combines body sensation techniques with mindfulness based interventions and helps mainly in overall acceptance. Our results are therefore in line with the findings of other studies where teaching acceptance in obesity is examined [26, 27]. Even more, patients’ acceptance seems to improve and remain stable over at least one year in the present study.

Our data not only show that increased skills of regulation of emotion may have had an influence on weight loss, but also on reducing psychosomatic symptoms and improving general wellbeing (i.e., reduction of depression levels and shame) which was a further main objective of the study. Regarding this, we demonstrated that psychosomatic and other psychological measures may have an influence on BMI. In accordance with the high somatic comorbidities in obesity (e.g. chronic pain, musculoskeletal or cardiovascular disorders [4, 28]) our study shows that somatization may lead to a relevant increase in BMI (by 0.06 kg/m^2^ in our study). Furthermore, an increase in interpersonal sensitivity and depression symptoms may also lead to an increase in BMI (by 0.24 kg/m^2^ and 0.05 kg/m^2^ in our study). In our sample, the average level of depressive symptoms was just below the clinical relevant cut-off of the SCL 90-R score system. Important aspects of a higher depression score are change in appetite [29] and social withdrawal [30, 31].

Evidence suggests that the reduction of emotional induced eating reflects lower levels of negative emotions [32, 33]. While our findings in the scale “negative affect” support this hypotheses, our results in the scale “negative affect total” are in contrast to this hypothesis, indicating that negative affect promotes weight gain, which is a surprising effect. However, an in depth analysis of the score system items “negative affect” and “negative affect in total” explain this difference: The scale “negative affect in total” includes in general more affective items and measures emotional states of a longer duration (e.g. behavior) than the scale “negative affect” (e.g. circumstance, actual emotional state). Individuals seem to be able to rapidly improve the regulation of an actual emotional state, but need more time to deal with complex long-term emotional situations such as depression. Patients with obesity seem to benefit from a shift in their goals after an initial weight reduction time [34]. Our findings give insight that it could be helpful to shift the patients’ goals from behaviour change towards better awareness of emotional change and emotion regulation skills. Still, due to the high comorbidity of obesity and depression [5, 6] it is not surprising that body weight may be linked to an increase in the presence of longer lasting and more complex negative emotions.

Addressing the influence of emotion regulation skills on BMI, we found that attention to emotion (BMI reduced by −0.15 per unit), awareness of bodily sensations (BMI reduced by −0.16 per unit) and emotional clarity (BMI increased by 0.36 per unit) are relevant outcome variables in our models. One interesting finding is a possible association between emotional clarity and an increase in BMI. A possible explanation lies in the application of a suitable psychological treatment [35]. According to Grawe et al. [35] a major step in psychological treatment is the “problem activation” where patients more intensively encounter the formerly neglected problems. This usually goes hand in hand with an increase in negative emotions. In light of the above-mentioned improvements in “body awareness” and “attention to emotions” we conclude that patients may have learned to understand how negative emotions originated, but initially they were not yet able to control them. As patients still lack other emotion regulation abilities, they fall back into former strategies, for instance emotional eating. In an evaluation of a weight loss intervention for individuals with emotional eating patterns, Goldbacher et al. [36] compared a behavioural change-only weight loss treatment with a specific enhanced-behavioural treatment with skills training for emotional eating. Patients lost a significant amount of weight and reduced emotional eating in both treatment groups during a 4 month intervention. This again illustrates that focusing on emotion regulation skills in a standardized behavioural weight loss intervention may help in weight reduction in obese individuals and that adding emotional-eating specific strategies may not provide additional benefits in the short term. Similar findings were found by Stapleton et al. (2016) [37] comparing an intervention in emotional freedom technique (EFT) with cognitive behavioral therapy. Also in this randomized controlled trial, both intervention groups had similar treatment effects regarding weight as well as psychological outcomes within a 12 months follow up. Our findings provide additional evidence that patients who are not yet able to adapt new emotion regulation skills in the short term, may yet be able to do so on the long term. This may result in improved general well-being and greater weight loss. However, such an association with weight loss was not found in this study. This observation is to some extent contradictory to research on emotional eating, promoting specific emotional eating skills to reduce weight [8].

Finally, our study did not show a difference between the group and the individual MOTP. We cannot accurately interpret these results further due to the difference between the two treatment programs.

Nevertheless some limitations to our study have to be considered. The main limitation in this study was lack of a control group. It is also noted that there were relatively few (only 9/38) positive associations found between measures of psychological and emotional status and weight change. Further, due to the complexity of obesity, not adequately represented by a single outcome and the naturalistic design of the study, we did not define a single outcome variable in the study but measured treatment outcome and showed treatment effectiveness by several variables of interest. There was a high rate of dropouts, which is comparable with other international studies [38]. Income and education were not controlled, which are known as relevant factors in obesity [39, 40]. However, due to the fact that the program was fully covered by health insurance these factors may not have influenced patient recruiting or outcome. We only included German speaking participants in the study. Furthermore, there were more women than men. As men and women seem to differ in emotional eating patterns [9] we might have a gender bias by having a mostly female sample with superior skills related to attention to emotions and emotion regulation. Additionally, it remains unclear in which aspects the body awareness and the behavioral focused psychological treatment were complementary, and in which aspects each treatment added specific effects. Also, we did not measure the persistence of change after the one year treatment. In general, longer term and controlled trials are needed to investigate these findings further.

## Conclusions

The current study demonstrates that an interdisciplinary MOTP was associated with weight reduction and improved psychological status. Psychological factors such as somatization, interpersonal sensitivity, attention to emotions, body-awareness of emotions, emotional clarity, depression, negative affect, disgust and shame were associated with influence on weight reduction. Therefore, behavioral and cognitive psychological interventions focussing on depression, emotion regulation and mindfulness-based body awareness therapy are potentially useful elements of a MOPT, which support successful lifestyle modifications and weight loss in obese individuals.

## Abbreviations

BMI: Body Mass Index (in kg/m^2^)
CBT: Cognitive Behavioral Therapy
MOTP: Multiprofessional Obesity Treatment Program
OOC: Obesity Outpatient Clinic
