# Erratum to: Evaluation of a multiprofessional, nonsurgical obesity treatment program: which parameters indicated life style changes and weight loss?

**DOI:** 10.1186/s40337-017-0167-x

**Published:** 2017-10-27

**Authors:** Irena Pjanic, Roland Müller, Markus Laimer, Niels Hagenbuch, Kurt Laederach, Zeno Stanga

**Affiliations:** 1 Department of Diabetes, Endocrinology, Clinical Nutrition and Metabolism, University of Bern and University Hospital of Bern, 3010 Bern, Switzerland; 2 Private statistician, Spiez, Switzerland; 3 Department of Department of Visceral Surgery and Medicine, University of Bern and University Hospital of Bern, 3010 Bern, Switzerland

## Erratum

Unfortunately, the original version of this article [1] contained an error. The first name of “Irena Pjanic” was omitted. The original article has been corrected.
